# Supplementary material for: Negative Electronic Friction and Non-Markovianity in Nonequilibrium Quantum Systems
Source: Nano Lett. 2026 Jun 12;26(24):8004–9. doi: 10.1021/acs.nanolett.6c01839 (PMC13307275; doi:10.1021/acs.nanolett.6c01839)
Supplement: Supplementary file 1 [file nl6c01839_si_001.pdf]

Supplemental Material: Negative Electronic Friction and Non-Markovianity in Nonequilibrium Quantum Systems

R. J. Preston,[1] S. L. Rudge,[1] D. S. Kosov,[2] and M. Thoss[1]

[1] *Institute of Physics, University of Freiburg, Hermann-Herder-Str. 3, D-79104 Freiburg, Germany*
[2] *College of Science and Engineering, James Cook University, Townsville, QLD, 4811, Australia*

I. MIXED QUANTUM-CLASSICAL METHODOLOGY

This section outlines the range of mixed quantum-classical approaches employed in this work. Beginning from a quantum framework, we first present the equations of motion for the vibrations that emerge under the classical limit. Next, we introduce the Ehrenfest and EFLD approaches via further approximations on the electronic forces. Although we provide an outline of these approaches for the sake of self-completeness, more detailed derivations can be found in Refs. [1, 2]. Note that we also restrict all derivations to a single vibrational coordinate, although a generalization to multiple coordinates is possible.

A. Classical Equation of Motion for the Vibrational Degrees of Freedom

In the following, it is natural to split the total Hamiltonian into an electronic and vibrational part,

$$H = H_{\text{el}}(\hat{x}(t)) + H_{\text{vib}}. \tag{1}$$

To connect with the Hamiltonian introduced in the main text, note that the electronic part contains all electronic degrees of freedom from the molecule and metal surface as well as the electronic-vibrational coupling,

$$H_{\text{el}}(\hat{x}(t)) = H_{\text{mol-el}}(\hat{x}(t)) + H_{\text{mol-leads}} + H_{\text{leads}} \tag{2}$$

$$H_{\text{vib}} = H_{\text{mol-vib}}. \tag{3}$$

As detailed in Ref.[1], one then writes the time evolution of the vibrational density operator, which is obtained by tracing out the electronic degrees of freedom from the total density operator,

$$\rho_{\text{vib}}(t) = \text{Tr}_{\text{el}} \left\{ \rho_{\text{tot}}(t) \right\}, \tag{4}$$

exactly in terms of the electronic Feynman-Vernon influence functional. Next, the vibrational coordinate is written in terms of the average vibrational trajectory, $x(t)$, and some quantum difference, $\hat{\delta}x(t)$,

$$\hat{x}(t) = x(t) + \hat{\delta}x(t) \tag{5}$$

and the influence functional is expanded to second order in both $\hat{\delta}x(t)$ and the electronic influence, yielding a Gaussian stochastic equation of motion for $x(t)$:

$$m\ddot{x} = -\partial_x U_{\text{vib}} + \langle F_{\text{el}} \rangle [x(t)] + f(t). \tag{6}$$

At this point, the time evolution is governed by the unperturbed harmonic vibrational potential, $U_{\text{vib}}(x)$, the path-dependent mean electronic force,

$$\langle F_{\text{el}} \rangle [x(t)] = \text{Tr}_{\text{el}} \left\{ F_{\text{el}}(x(t)) \rho_{\text{el}}(t) \right\}, \tag{7}$$

and a Gaussian electronic stochastic force, $f(t)$, with correlation function,

$$D(t, t') = \langle f(t) f(t') \rangle = \text{Tr}_{\text{el}} \left\{ \left[F^I_{\text{el}}(x(t)), F^I_{\text{el}}(x(t')) \right]_+ \rho_{\text{el}}(t_0) \right\}. \tag{8}$$

Here, we have introduced the electronic force operator,

$$F_{\text{el}}(x(t)) = -\partial_x H_{\text{el}}(x(t)), \tag{9}$$

evaluated instantaneously at the classical vibrational coordinate $x(t)$. We have also introduced the interaction picture with respect to the electronic degrees of freedom,

$$F^I_{\text{el}}(x(t)) = U^\dagger_{\text{el}}(t, t_0) F_{\text{el}}(x(t)) U_{\text{el}}(t, t_0), \tag{10}$$

where the time-evolution operator is

$$U_{\text{el}}(t, t_0) = \mathcal{T} \exp \left[-i \int_{t_0}^{t} d\tau H_{\text{el}}(x(\tau)) \right]. \tag{11}$$

From this, one sees that the electronic density matrix at time t,

$$\rho_{\rm el}(t) = U_{\rm el}(t,t_0)\rho_{\rm el}(t_0)U^{\dagger}_{\rm el}(t,t_0), \tag{12}$$

is actually a functional of the vibrational trajectory: $\rho_{\rm el}(t) = \rho_{\rm el}[x(t)]$.

At this point, no further approximations have been made beyond the assumption that quantum deviations from the average vibrational trajectory are small and the electronic influence is Gaussian. Physically, this may be satisfied for molecules where the vibrational degrees of freedom represent motion of the heavy atomic nuclei, which is much slower than the fast time evolution of the electronic degrees of freedom, or for systems with weak electronic-vibrational coupling. This is especially true for harmonic vibrational potentials, where the expansion of the unperturbed vibrations in $\delta x(t)$ would terminate naturally at second order, and the only higher-order quantum corrections arise from the influence of the quantum electronic degrees of freedom.

B. Ehrenfest Dynamics

The Ehrenfest equation of motion is then obtained by neglecting the stochastic force in Eq.(6) entirely, such that the classical vibrations respond only to the mean influence of the electronic environment,

$$m\ddot{x} = -\partial_x U_{\rm vib} + \langle F_{\rm el}\rangle[x(t)]. \tag{13}$$

Often, $\langle F_{\rm el}\rangle[x(t)]$ is referred to as the Ehrenfest force. Since $\langle F_{\rm el}\rangle[x(t)]$ contains a weighted average over all electronic adiabats, it can be used to treat highly nonadiabatic problems, even for large electronic-vibrational coupling. However, its accuracy relies on the assumption that all electronic adiabats have a similar shape, such that their influence is well captured by a weighted average. While this is generally a good approximation in the context of molecules interacting with metal surfaces due to the continuum of states in the metal, it is also a completely deterministic and coherent approach, as it neglects the stochastic force. Consequently, it excludes irreversible energy transfer between the vibrational and electronic degrees of freedom, resulting in an absence of critical dissipative features such as detailed balance and Joule heating [3, 4].

In this work, the Ehrenfest force is calculated via NEGFs according to the procedure detailed in Ref.[5]. When doing Ehrenfest dynamics for the model under consideration, trajectories either decay to a state of zero excitation due to a lack of Joule heating, or reach a limit cycle in the vibrational steady-state that is independent of the initial conditions. As such, observables are calculated by time-averaging over the vibrational steady-state of a single trajectory. Expectation values of observables are obtained via the method outlined in Sec. I D.

C. Electronic Friction and Langevin Dynamics

In contrast to the Ehrenfest approach, the electronic friction approach retains detailed balance and other statistical effects in the limit of weak nonadiabaticity [1, 6, 7]. The critical assumption is that there is a clear timescale separation between fast-relaxing electronic degrees of freedom and slow vibrational degrees of freedom, such that the mean electronic force can be expanded in powers of the vibrational velocity,

$$\langle F_{\rm el}\rangle(t) \approx \langle F_{\rm el}(t)\rangle^{\rm ss} - \int_{-\infty}^{\infty} d\tau\, \gamma(t,\tau)\dot{x}(\tau). \tag{14}$$

Here, we have introduced the zeroth-order or adiabatic contribution to the mean force,

$$F^{\rm ad}_{\rm el}(x(t)) = \langle F_{\rm el}(x(t))\rangle^{\rm ss} = \,{\rm Tr}_{\rm el}\left\{F_{\rm el}(x(t))\rho^{\rm ss}_{\rm el}(x(t))\right\}, \tag{15}$$

which is calculated at a fixed vibrational frame $x(t)$ for $\dot{x}(t) = 0$, assuming that the electronic degrees of freedom relax instantaneously to a stationary state, $\rho^{\rm ss}_{\rm el}(x(t))$, where $\mathcal{L}_{\rm el}(x)\rho^{\rm ss}_{\rm el}(x) = 0$ and $\mathcal{L}_{\rm el}(x) = -i\left[H_{\rm el}(x), \ldots\right]$.

In contrast, the first-order term represents a nonadiabatic correction that incorporates the dissipation of vibrational energy due to electron-hole pair (EHP) formation in the leads, which is contained in the electronic friction kernel:

$$\gamma(t,\tau) = -\theta(t-\tau)e^{-\delta(t-\tau)}{\rm Tr}_{\rm el}\left\{F_{\rm el}(t)U_{\rm el}(t,\tau)\partial_x\rho^{\rm ss}_{\rm el}(x(t))U^{\dagger}_{\rm el}(t,\tau)\right\}. \tag{16}$$

In Eq.(16), the $\theta(t-\tau)$ component ensures causality, while the $e^{-\delta(t-\tau)}$ ensures nuclear driving on a timescale slower than the decay of $\gamma(t,\tau)$. If this decay is determined by $\tau_{\rm el}$, then the appropriate limit is $\delta \ll \tau^{-1}_{\rm el}$.

Considering that the electronic friction kernel is calculated in the limit of slow vibrational driving, a further quasi-stationary approximation is often employed, such that the vibrational coordinate is approximately stationary over $\tau_{\rm el}$ and $x(\tau) \approx x(t)$. Physically, the electrons forget the absolute time and depend only on the relative time $t-\tau$, which simplifies the time-evolution operator, $U_{\rm el}(t,\tau) \to e^{-iH_{\rm el}(x(t))(t-\tau)}$. Consequently, in this limit, the electronic friction tensor and correlation function of the stochastic force take the form

$$\gamma(x(t), t-\tau) = -\theta(t-\tau)e^{-\delta(t-\tau)}{\rm Tr}_{\rm el}\left\{F_{\rm el}(t)e^{-iH_{\rm el}(x(t))(t-\tau)}\partial_x\rho^{\rm ss}_{\rm el}(x(t))e^{iH_{\rm el}(x(t))(t-\tau)}\right\}. \tag{17}$$

$$D(x(t), t-\tau) = {\rm Tr}_{\rm el}\left\{F_{\rm el}(t)e^{-iH_{\rm el}(x(t))(t-\tau)}\left(\delta F_{\rm el}(t)\rho^{\rm ss}_{\rm el}(x(t)) + \rho^{\rm ss}_{\rm el}(x(t))\delta F_{\rm el}(t)\right)e^{iH_{\rm el}(x(t))(t-\tau)}\right\}, \tag{18}$$

where $\delta F_{\rm el}(t) = F_{\rm el}(t) - F^{\rm ad}_{\rm el}(t)$. The classical vibrations now follow a generalized Langevin equation (GLE),

$$m\ddot{x} = -\partial_x U_{\rm vib} + F^{\rm ad}_{\rm el}(x) - \int_0^t d\tau\, \gamma(x(t), t-\tau)\dot{x}(\tau) + f(t), \tag{19}$$

which corresponds to Eq.(1) in the main text. Even in the quasi-stationary limit, the GLE is still non-Markovian and the stochastic force still has colored noise. As a result, a further Markovian approximation is often made, which assumes that $\dot{x}(\tau)$ does not change appreciably over $\tau_{\rm el}$, such that $\dot{x}(\tau) \approx \dot{x}(t)$ and

$$m\ddot{x} = -\partial_x U_{\rm vib} + F^{\rm ad}_{\rm el}(x) - \underbrace{\int_0^t d\tau\, \gamma(x(t), t-\tau)}_{=\tilde{\gamma}(x,0)}\dot{x}(t) + f(t). \tag{20}$$

Here, we can identify $\tilde{\gamma}(x,0)$ as the zero-frequency component of the electronic friction spectrum,

$$\tilde{\gamma}(x,\omega) = \int d\tau e^{i\omega\tau}\gamma(x,\tau), \tag{21}$$

which is the Markovian electronic friction coefficient. Furthermore, in the Markovian limit, the stochastic force is characterized by white noise,

$$D(t,t') = \tilde{D}(x,0)\delta(t-t'), \tag{22}$$

such that the GLE in Eq.(19) reduces to a Markovian Langevin equation,

$$m\ddot{x} = -\partial_x U_{\rm vib} + F^{\rm ad}_{\rm el}(x) - \tilde{\gamma}(x,0)\dot{x} + f(t). \tag{23}$$

Unlike Ehrenfest dynamics, detailed balance is built directly into Eq.(23) and Eq.(19), so it captures important statistical processes such as Joule heating. In equilibrium, for example, the finite-frequency electronic friction coefficient and the correlation of the stochastic force satisfy the quantum flucutation-dissipation theorem (FDT) [1]:

$$\tilde{D}(x,\omega) = \omega\coth\left(\frac{\omega}{2k_BT}\right){\rm Re}\left\{\tilde{\gamma}(x,\omega)\right\}, \tag{24}$$

which reduces to the classical FDT in the Markovian limit

$$\tilde{D}(x,0) = k_BT\tilde{\gamma}(x,0). \tag{25}$$

As with the Ehrenfest approach, in this work we calculate the electronic forces via NEGFs, with exact expressions given in Sec. II.

1. *Energy Dissipation of the Electronic Friction*

To understand the dissipative or driving nature of the non-Markovian electronic friction force, we consider the instantaneous power dissipated by the vibrational mode to it:

$$P_{\rm diss.} = \frac{dW_{\rm fric.}}{dt} = -\dot{x}(t)\int_{-\infty}^{\infty} d\tau\, \gamma(x(t), t-\tau)\dot{x}(\tau), \tag{26}$$

where $P_{\text{diss.}} \geq 0$ indicates that the electronic friction has an overall damping effect. Now, in the near-adiabatic limit, the total dissipated energy is determined by the long-time average power at a particular coordinate,

$$\begin{aligned}
\bar{P}_{\text{diss.}}(x) &= \int_{-\infty}^{\infty} dt\, P_{\text{diss.}} && (27)\\
&= -\int_{-\infty}^{\infty} dt\, \dot{x}(t) \left(\frac{1}{2\pi} \int_{-\infty}^{\infty} d\omega\, e^{-i\omega t} \tilde{\gamma}(x,\omega) \tilde{\dot{x}}(\omega) \right) && (28)\\
&= -\frac{1}{2\pi} \int_{-\infty}^{\infty} d\omega\, \underbrace{|\tilde{\dot{x}}(\omega)|^2 \tilde{\gamma}(x,\omega)}_{\bar{P}_{\text{diss.}}(x,\omega)}, && (29)
\end{aligned}$$

where we have identified the power density $\bar{P}_{\text{diss.}}(x,\omega)$ and used the Fourier transform of the convolution in Eq.(19),

$$\int_{-\infty}^{\infty} dt\, e^{i\omega t} \int_0^t d\tau\, \gamma(x(t), t-\tau)\dot{x}(\tau) = \tilde{\gamma}(x,\omega)\tilde{\dot{x}}(\omega). \tag{30}$$

For real velocities, negative and positive frequencies are related via $\tilde{\dot{x}}(\omega)^* = \tilde{\dot{x}}(-\omega)$, such that $|\tilde{\dot{x}}(-\omega)|^2 = |\tilde{\dot{x}}(\omega)|^2$. Furthermore, we can separate the Fourier transform of the friction into real and imaginary components, $\tilde{\gamma}(x,\omega) = \text{Re}\{\tilde{\gamma}(x,\omega)\} + i\text{Im}\{\tilde{\gamma}(x,\omega)\}$. Given that the friction kernel is causal, $\gamma(t<0)=0$, $\tilde{\gamma}^*(x,\omega) = \tilde{\gamma}(x,-\omega)$ and $\text{Im}\{\tilde{\gamma}(x,-\omega)\} = -\text{Im}\{\tilde{\gamma}(x,\omega)\}$. Consequently, the imaginary component of the friction does not contribute to the dissipated power, and the power density relies only on the real part of the friction kernel:

$$\bar{P}_{\text{diss.}}(x,\omega) = |\tilde{\dot{x}}(\omega)|^2 \text{Re}\{\gamma(x,\omega)\}. \tag{31}$$

From the form of Eq.(31) and Eq.(29), we see that the electronic friction force has dissipative (driving) effect at coordinate x and frequency ω if $\text{Re}\{\gamma(x,\omega)\} > 0$ ($\text{Re}\{\gamma(x,\omega)\} < 0$).

D. Numerical Details and Observables in MQC Methods

In all numerical simulations, we work in the mass- and frequency-scaled units introduced in the main text, $\{x,p\} \to \{x\sqrt{m\Omega}, p/\sqrt{m\Omega}\}$.

In the Ehrenfest dynamics simulations, observables are calculated as a time average over the limit cycle observed in the vibrational stready state. The expectation value of a given observable, O, is thus computed via

$$\langle O \rangle = \frac{1}{t_{\text{fin}} - t_{\text{ss}}} \int_{t_{\text{ss}}}^{t_{\text{fin}}} dt\, O(t), \tag{32}$$

where t_{ss} is the time at which the vibrations reach the limit cycle, and t_{fin} is the end of the trajectory.

Rather than propagating the Markovian Langevin equation to find the vibrational steady state, we opt to instead compute the vibrational steady state distribution directly from the corresponding Markovian Fokker-Planck equation, which is an equivalent description of the same stochastic dynamics [8, 9]. Observables are then computed via

$$\langle O \rangle = \int_{-\infty}^{\infty} dx \int_{-\infty}^{\infty} dv O(x,v) P_{\text{ss}}(x,v), \tag{33}$$

where $P_{\text{ss}}(x,v)$ is the vibrational steady state distribution in the space of x and v. The results obtained by this approach were also compared to results obtained by propagating the Markovian Langevin equation directly via a Trotter decomposition scheme (for details, see Refs.[10, 11]) to ensure agreement.

II. ELECTRONIC FORCES IN TERMS OF NONEQUILIBRIUM GREEN'S FUNCTIONS

In this section, we derive exact expressions for the electronic forces in the NEGF formalism. In the following derivations, we explicitly consider the case of a single vibrational degree of freedom. We additionally assume that the molecule-lead couplings are independent of that vibrational degree of freedom.

A. Green's function theory

The basic building blocks in our derivation are nonadiabatic exact retarded, advanced, lesser, and greater Green's functions in the electronic space of the molecular bridge, calculated with a fully time-dependent Hamiltonian along a given trajectory $x(t)$:

$$G^R_{ij}(t,t') = -i\theta(t-t')\langle\{d_i(t), d_j^\dagger(t')\}_+\rangle, \qquad G^A_{ij}(t,t') = \left(G^R_{ji}(t',t)\right)^\dagger, \tag{34}$$

$$G^<_{ij}(t,t') = i\langle d_i(t)d_j^\dagger(t')\rangle, \qquad G^>_{ij}(t,t') = -i\langle d_j^\dagger(t')d_i(t)\rangle. \tag{35}$$

The Green's functions evolve according to the Keldysh-Kadanoff-Baym equations of motion,

$$\left(i\frac{\partial}{\partial t}I - h(x(t))\right)G^{A/R}(t,t') = I\delta(t-t') + \int_{-\infty}^{\infty} dt_1 \Sigma^{A/R}(t,t_1)G^{A/R}(t_1,t'), \tag{36}$$

$$\left(i\frac{\partial}{\partial t}I - h(x(t))\right)G^{</>}(t,t') = \int_{-\infty}^{\infty} dt_1\left(\Sigma^{</>}(t,t_1)G^A(t_1,t') + \Sigma^R(t,t_1)G^{</>}(t_1,t')\right), \tag{37}$$

where the Green's functions, electronic Hamiltonian for the molecular bridge h, and self-energies Σ are written as matrices in the single-particle basis for the molecular bridge. The electronic Hamiltonian for the molecular bridge is then defined as

$$H_{\text{mol-el}}(x) = \sum_{ij} h_{ij}(x)d_i^\dagger d_j. \tag{38}$$

The present derivation is for general $h(x)$ and is not specific to the model presented in the main text.

We introduce the Wigner time coordinates,

$$T = \frac{t+t'}{2}, \qquad \tau = t - t', \tag{39}$$

where T is associated with the classical vibration timescale and τ is associated with the electronic relaxation timescale. By taking the Fourier transform of each time coordinate individually, $\tau \to \epsilon$ and $T \to \omega$, we obtain

$$\left(\epsilon + \frac{\omega}{2}\right)\tilde{G}^{A/R}(\omega,\epsilon) - \int_{-\infty}^{\infty}\frac{d\omega'}{2\pi}\tilde{h}(\omega')\tilde{G}^{A/R}(\omega-\omega',\epsilon-\frac{\omega'}{2}) = 2\pi\delta(\omega)I + \tilde{\Sigma}^{A/R}(\epsilon+\frac{\omega}{2})\tilde{G}^{A/R}(\omega,\epsilon), \tag{40}$$

$$\left(\epsilon + \frac{\omega}{2}\right)\tilde{G}^{</>}(\omega,\epsilon) - \int_{-\infty}^{\infty}\frac{d\omega'}{2\pi}\tilde{h}(\omega')\tilde{G}^{</>}(\omega-\omega',\epsilon-\frac{\omega'}{2}) = \tilde{\Sigma}^{</>}(\epsilon+\frac{\omega}{2})\tilde{G}^{A}(\omega,\epsilon) + \tilde{\Sigma}^{R}(\epsilon+\frac{\omega}{2})\tilde{G}^{</>}(\omega,\epsilon), \tag{41}$$

where

$$\tilde{G}(\omega,\epsilon) = \int_{-\infty}^{\infty} dT e^{i\omega T}\int_{-\infty}^{\infty} d\tau e^{i\epsilon\tau}G(t,t'). \tag{42}$$

In Eq.(40) and Eq.(41), the influence of the vibration on the electronic dynamics is contained within a convolution over the vibrational frequency. In an exact treatment of the electronic dynamics, this term has components:

$$\begin{aligned}\int_{-\infty}^{\infty}\frac{d\omega'}{2\pi}\tilde{h}(\omega')\tilde{G}(\omega-\omega',\epsilon-\frac{\omega'}{2}) = \;& h_{\text{ad.}}(x)\tilde{G}(\omega,\epsilon) + \int_{-\infty}^{\infty}\frac{d\omega'}{2\pi}\left(\frac{-\omega'}{2}\right)\frac{dh_{\text{ad}}(x)}{dx}\tilde{x}(\omega')\frac{\partial\tilde{G}(\omega-\omega',\epsilon)}{\partial\epsilon} \\ &+ \int_{-\infty}^{\infty}\frac{d\omega'}{2\pi}\left(\frac{-\omega'}{2}\right)^2\frac{dh_{\text{ad}}(x)}{dx}\tilde{x}(\omega')\frac{\partial^2\tilde{G}(\omega-\omega',\epsilon)}{\partial\epsilon^2} + \dots \\ &+ \int_{-\infty}^{\infty}\frac{d\omega'}{2\pi}\int_{-\infty}^{\infty}\frac{d\omega''}{2\pi}\left(\frac{-\omega'}{2}\right)\left(\frac{-\omega''}{2}\right)\frac{d^2h_{\text{ad}}(x)}{dx^2}\tilde{x}(\omega')\tilde{x}(\omega'')\frac{\partial^2\tilde{G}(\omega-\omega'-\omega'',\epsilon)}{\partial\epsilon^2} \\ &+ \dots. \end{aligned} \tag{43}$$

We use $h_{\text{ad.}}(x)$ to denote the Hamiltonian evaluated instantaneously at x, where the frequency dependence is now contained within $\tilde{x}$. Motivated by the form of Eq.(43), we propose the following ansätze,

$$\tilde{h}(\omega) = 2\pi h_{\text{ad.}}(x)\delta(\omega) + \frac{dh_{\text{ad.}}(x)}{dx}\tilde{x}(\omega), \qquad \tilde{G}(\omega,\epsilon) = 2\pi\delta(\omega)\tilde{G}_{(0)}(x,\epsilon) + \tilde{G}_{(1)}(x,\omega,\epsilon), \tag{44}$$

where the Hamiltonian is expanded to first order in the parametric time dependence induced by the nuclear trajectory $x(t)$, and the Green's function is correspondingly expanded to first order in this time-dependent perturbation, so that $\tilde{G}_{(0)}$ treats the dynamics adiabatically and $\tilde{G}_{(1)}$, which is linear in $\frac{dh_{\text{ad.}}(x)}{dx}\tilde{x}$, accounts for the linear response of the Green's function to the perturbation. By employing these ansätze and collecting terms of the same order in Eq.(40) and Eq.(41), we can derive the adiabatic Green's functions in the standard form,

$$\tilde{G}^{A/R}_{(0)}(x,\epsilon) = \Big(\epsilon I - h(x) - \tilde{\Sigma}^{A/R}(\epsilon)\Big)^{-1}, \qquad \tilde{G}^{</>}_{(0)}(x,\epsilon) = \tilde{G}^{R}_{(0)}(x,\epsilon)\tilde{\Sigma}^{</>}_{(0)}(\epsilon)\tilde{G}^{A}_{(0)}(x,\epsilon), \tag{45}$$

while the first order Green's functions are found to be

$$\tilde{G}^{A/R}_{(1)}(x,\omega,\epsilon) = \tilde{x}(\omega)\tilde{G}^{A/R}_{(0)}(x,\epsilon+\frac{\omega}{2})\frac{dh_{\text{ad.}}(x)}{dx}\tilde{G}^{A/R}_{(0)}(x,\epsilon-\frac{\omega}{2}), \tag{46}$$

$$\tilde{G}^{</>}_{(1)}(x,\omega,\epsilon) = \tilde{x}(\omega)\Big(\tilde{G}^{R}_{(0)}(x,\epsilon+\frac{\omega}{2})\frac{dh_{\text{ad.}}(x)}{dx}\tilde{G}^{</>}_{(0)}(x,\epsilon-\frac{\omega}{2}) + \tilde{G}^{</>}_{(0)}(x,\epsilon+\frac{\omega}{2})\frac{dh_{\text{ad.}}(x)}{dx}\tilde{G}^{A}_{(0)}(x,\epsilon-\frac{\omega}{2})\Big). \tag{47}$$

The self-energies are defined as

$$\tilde{\Sigma}^{<}(\epsilon) = i\sum_{\alpha\in\{L,R\}} f_\alpha(\epsilon)\Gamma_\alpha(\epsilon), \qquad \tilde{\Sigma}^{>}(\epsilon) = -i\sum_{\alpha\in\{L,R\}}(1-f_\alpha(\epsilon))\Gamma_\alpha(\epsilon), \tag{48}$$

$$\tilde{\Sigma}^{A}(\epsilon) = \sum_{\alpha\in\{L,R\}}\Big(\Lambda_\alpha(\epsilon) + \frac{i}{2}\Gamma_\alpha(\epsilon)\Big), \qquad \tilde{\Sigma}^{A}(\epsilon) = \sum_{\alpha\in\{L,R\}}\Big(\Lambda_\alpha(\epsilon) + \frac{i}{2}\Gamma_\alpha(\epsilon)\Big). \tag{49}$$

We explicitly take the wide-band limit in our calculations so that $\Lambda_\alpha(\epsilon) = 0$ and $\Gamma_\alpha(\epsilon) \to \Gamma_\alpha$.

B. Non-Markovian Langevin Coefficients

The non-Markovian Langevin coefficients are now derived in terms of the adiabatic and first-order Green's functions.

1. *Adiabatic force and electronic friction tensor*

The mean electronic force is

$$\langle F_{\text{el}}\rangle(t) = i\text{Tr}\Big\{\frac{dh_{\text{ad.}}(x)}{dx}G^{<}(t,t)\Big\}. \tag{50}$$

Fourier transforming to the (ω,ϵ) space yields

$$\langle \tilde{F_{\text{el}}}\rangle(\omega) = \frac{i}{2\pi}\int_{-\infty}^{\infty} d\epsilon\text{Tr}\Big\{\frac{dh_{\text{ad.}}(x)}{dx}\tilde{G}^{<}(\omega,\epsilon)\Big\}. \tag{51}$$

By applying our Green's function ansatz in Eq.(44), the mean force is decomposed into an adiabatic force and a first-order frictional force,

$$\langle \tilde{F_{\text{el}}}\rangle(\omega) \approx \tilde{f}_{(0)}(x,\omega) + \tilde{f}_{(1)}(x,\omega). \tag{52}$$

The adiabatic force is calculated as

$$\tilde{f}_{(0)}(x) = \frac{i}{2\pi}\int_{-\infty}^{\infty} d\epsilon\text{Tr}\Big\{\frac{dh_{\text{ad.}}(x)}{dx}\tilde{G}^{<}_{(0)}(x,\epsilon)\Big\}. \tag{53}$$

The frictional force is calculated via the first-order Green's function,

$$\tilde{f}_{(1)}(x,\omega) = \frac{i}{2\pi}\int_{-\infty}^{\infty} d\epsilon \mathrm{Tr}\Big\{\frac{dh_{\mathrm{ad.}}(x)}{dx}\tilde{G}^{<}_{(1)}(x,\omega,\epsilon)\Big\} \tag{54}$$

$$= \frac{i\tilde{x}(\omega)}{2\pi}\int_{-\infty}^{\infty} d\epsilon\ \mathrm{Tr}\Big\{\frac{dh_{\mathrm{ad.}}(x)}{dx}\tilde{G}^{R}_{(0)}(x,\epsilon+\frac{\omega}{2})\frac{dh_{\mathrm{ad.}}(x)}{dx}\tilde{G}^{<}_{(0)}(x,\epsilon-\frac{\omega}{2}) + \frac{dh_{\mathrm{ad.}}(x)}{dx}\tilde{G}^{<}_{(0)}(x,\epsilon+\frac{\omega}{2})\frac{dh_{\mathrm{ad.}}(x)}{dx}\tilde{G}^{A}_{(0)}(x,\epsilon-\frac{\omega}{2})\Big\} \tag{55}$$

$$= i\omega\tilde{x}(\omega)\tilde{\gamma}(x,\omega), \tag{56}$$

where we have defined the power spectrum of the electronic friction kernel as

$$\tilde{\gamma}(x,\omega) = \frac{1}{2\pi\omega}\int_{-\infty}^{\infty} d\epsilon\ \mathrm{Tr}\Big\{\frac{dh_{\mathrm{ad.}}(x)}{dx}\tilde{G}^{R}_{(0)}(x,\epsilon+\frac{\omega}{2})\frac{dh_{\mathrm{ad.}}(x)}{dx}\tilde{G}^{<}_{(0)}(x,\epsilon-\frac{\omega}{2}) + \frac{dh_{\mathrm{ad.}}(x)}{dx}\tilde{G}^{<}_{(0)}(x,\epsilon+\frac{\omega}{2})\frac{dh_{\mathrm{ad.}}(x)}{dx}\tilde{G}^{A}_{(0)}(x,\epsilon-\frac{\omega}{2})\Big\}. \tag{57}$$

By taking the inverse Fourier transform of Eq.(56), we obtain the frictional force in the time domain,

$$f_{(1)}(x,t) = -\int_{-\infty}^{\infty} d\tau\gamma(x,t-\tau)\dot{x}(\tau), \tag{58}$$

with electronic friction kernel,

$$\gamma(x,\tau) = \int_{-\infty}^{\infty}\frac{d\omega}{2\pi}e^{-i\omega\tau}\tilde{\gamma}(x,\omega). \tag{59}$$

2. *Stochastic force correlation function*

The stochastic force is treated as a Gaussian process so that it is quantified by its first two moments,

$$\langle f(t)\rangle = 0, \qquad \langle f(t)f(t')\rangle = D(t,t'), \tag{60}$$

where we use $\langle\dots\rangle$ to denote an unconditioned average over the realizations of the stochastic force process along a particular vibrational trajectory. Since the stochastic force generally obeys colored noise statistics, the conditional mean of $f(t)$ given a particular realization of the stochastic force history along the nuclear trajectory is usually non-zero. The zero-mean condition above refers only to the unconditional average over realizations of the stochastic force. The two-time correlation function for the stochastic force, which is also a functional of the nuclear trajectory, is computed in terms of nonequilibrium Green's functions as

$$D(t,t') = \langle f(t)f(t')\rangle = \mathrm{ReTr}\Big\{\frac{dh_{\mathrm{ad.}}(x)}{dx}G^{>}(t,t')\frac{dh_{\mathrm{ad.}}(x)}{dx}G^{<}(t',t)\Big\}. \tag{61}$$

Fourier transforming to the (ω,ϵ) space yields

$$\tilde{D}(x,\omega,\epsilon) = \frac{1}{8\pi^2}\int_{-\infty}^{\infty} d\epsilon'\int_{-\infty}^{\infty} d\omega'\Big(\frac{dh_{\mathrm{ad.}}(x)}{dx}\tilde{G}^{>}(\omega',\epsilon+\epsilon')\frac{dh_{\mathrm{ad.}}(x)}{dx}\tilde{G}^{<}(\omega-\omega',\epsilon') + \frac{dh_{\mathrm{ad.}}(x)}{dx}\tilde{G}^{<}(\omega',\epsilon+\epsilon')\frac{dh_{\mathrm{ad.}}(x)}{dx}\tilde{G}^{>}(\omega-\omega',\epsilon')\Big). \tag{62}$$

We compute the Green's functions adiabatically to obtain

$$\tilde{D}(x,\omega) = \frac{1}{4\pi}\mathrm{Tr}\int_{-\infty}^{\infty} d\epsilon\Big(\frac{dh_{\mathrm{ad.}}(x)}{dx}\tilde{G}^{>}_{(0)}(x,\epsilon+\frac{\omega}{2})\frac{dh_{\mathrm{ad.}}(x)}{dx}\tilde{G}^{<}_{(0)}(x,\epsilon-\frac{\omega}{2}) + \frac{dh_{\mathrm{ad.}}(x)}{dx}\tilde{G}^{<}_{(0)}(x,\epsilon+\frac{\omega}{2})\frac{dh_{\mathrm{ad.}}(x)}{dx}\tilde{G}^{>}_{(0)}(x,\epsilon-\frac{\omega}{2})\Big). \tag{63}$$

C. Fluctuation-dissipation relation

We now demonstrate that Eq.(63) and the real part of Eq.(57) satisfy the fluctuation-dissipation relation in chemical and thermal equilibrium. The real part of the electronic friction spectrum can be expressed as

$$\mathrm{Re}\Big\{\tilde{\gamma}(x,\omega)\Big\} = \frac{1}{4\pi\omega}\int_{-\infty}^{\infty} d\epsilon \mathrm{Tr}\Big\{ \frac{dh_{\mathrm{ad.}}(x)}{dx}\tilde{G}^{>}_{(0)}(x,\epsilon+\frac{\omega}{2})\frac{dh_{\mathrm{ad.}}(x)}{dx}\tilde{G}^{<}_{(0)}(x,\epsilon-\frac{\omega}{2}) - \frac{dh_{\mathrm{ad.}}(x)}{dx}\tilde{G}^{<}_{(0)}(x,\epsilon+\frac{\omega}{2})\frac{dh_{\mathrm{ad.}}(x)}{dx}\tilde{G}^{>}_{(0)}(x,\epsilon-\frac{\omega}{2})\Big\}. \tag{64}$$

It is useful to now introduce the spectral function of the molecular bridge hybridized with the α lead as

$$A_\alpha(x,\epsilon) = \tilde{G}^{R}_{(0)}(x,\epsilon)\Gamma_\alpha \tilde{G}^{A}_{(0)}(x,\epsilon), \tag{65}$$

The Green's functions can then be expressed according to

$$\tilde{G}^{<}_{(0)}(x,\epsilon) = i\sum_{\alpha\in\{L,R\}} f_\alpha(\epsilon)A_\alpha(x,\epsilon), \qquad \tilde{G}^{>}_{(0)} = -i\sum_{\alpha\in\{L,R\}}(1-f_\alpha(\epsilon))A_\alpha(x,\epsilon). \tag{66}$$

The real part of the friction power spectrum and the stochastic force correlation function then take the form,

$$\mathrm{Re}\Big\{\tilde{\gamma}(x,\omega)\Big\} = \frac{1}{4\pi\omega}\int_{-\infty}^{\infty} d\epsilon \sum_{\alpha\beta\in\{L,R\}} \mathrm{Tr}\Big\{ f_\beta(\epsilon-\frac{\omega}{2})\Big(1-f_\alpha(\epsilon+\frac{\omega}{2})\Big)\frac{dh_{\mathrm{ad.}}(x)}{dx}A_\alpha(x,\epsilon+\frac{\omega}{2})\frac{dh_{\mathrm{ad.}}(x)}{dx}A_\beta(x,\epsilon-\frac{\omega}{2}) - f_\alpha(\epsilon+\frac{\omega}{2})\Big(1-f_\beta(\epsilon-\frac{\omega}{2})\Big)\frac{dh_{\mathrm{ad.}}(x)}{dx}A_\alpha(x,\epsilon+\frac{\omega}{2})\frac{dh_{\mathrm{ad.}}(x)}{dx}A_\beta(x,\epsilon-\frac{\omega}{2})\Big\}, \tag{67}$$

$$\tilde{D}(x,\omega) = \frac{1}{4\pi}\int_{-\infty}^{\infty} d\epsilon \sum_{\alpha\beta\in\{L,R\}} \mathrm{Tr}\Big\{\Big(f_\beta(\epsilon-\frac{\omega}{2})\Big(1-f_\alpha(\epsilon+\frac{\omega}{2})\Big)\frac{dh_{\mathrm{ad.}}(x)}{dx}A_\alpha(x,\epsilon+\frac{\omega}{2})\frac{dh_{\mathrm{ad.}}(x)}{dx}A_\beta(x,\epsilon-\frac{\omega}{2}) + f_\alpha(\epsilon+\frac{\omega}{2})\Big(1-f_\beta(\epsilon-\frac{\omega}{2})\Big)\frac{dh_{\mathrm{ad.}}(x)}{dx}A_\alpha(x,\epsilon+\frac{\omega}{2})\frac{dh_{\mathrm{ad.}}(x)}{dx}A_\beta(x,\epsilon-\frac{\omega}{2})\Big)\Big\}. \tag{68}$$

Now, we assume chemical and thermal equilibrium such that $\mu_L = \mu_R$ and $T_L = T_R = T$, so that $f_L(\epsilon) = f_R(\epsilon)$. The above expressions then simplify to

$$\mathrm{Re}\Big\{\tilde{\gamma}(x,\omega)\Big\} = \frac{1}{4\pi\omega}\int_{-\infty}^{\infty} d\epsilon \sum_{\alpha\beta\in\{L,R\}} \Big(f(\epsilon-\frac{\omega}{2})f(\epsilon+\frac{\omega}{2})e^{\frac{\epsilon-\mu}{k_BT}}\Big[e^{\frac{\omega}{2k_BT}} - e^{-\frac{\omega}{2k_BT}}\Big]\Big) \times \mathrm{Tr}\Big\{\frac{dh_{\mathrm{ad.}}(x)}{dx}A_\alpha(x,\epsilon+\frac{\omega}{2})\frac{dh_{\mathrm{ad.}}(x)}{dx}A_\beta(x,\epsilon-\frac{\omega}{2})\Big\}, \tag{69}$$

$$\tilde{D}(x,\omega) = \frac{1}{4\pi}\mathrm{Tr}\Big[\int_{-\infty}^{\infty} d\epsilon \sum_{\alpha\beta\in\{L,R\}} \Big(f(\epsilon-\frac{\omega}{2})f(\epsilon+\frac{\omega}{2})e^{\frac{\epsilon-\mu}{k_BT}}\Big[e^{\frac{\omega}{2k_BT}} + e^{-\frac{\omega}{2k_BT}}\Big]\Big) \times \mathrm{Tr}\Big\{\frac{dh_{\mathrm{ad.}}(x)}{dx}A_\alpha(x,\epsilon+\frac{\omega}{2})\frac{dh_{\mathrm{ad.}}(x)}{dx}A_\beta(x,\epsilon-\frac{\omega}{2})\Big\}. \tag{70}$$

By noting that

$$\coth(x) = \frac{e^x + e^{-x}}{e^x - e^{-x}}, \tag{71}$$

we directly obtain the fluctuation-dissipation relation,

$$\tilde{D}(x,\omega) = \omega\mathrm{Re}\Big\{\tilde{\gamma}(x,\omega)\Big\}\coth\frac{\omega}{2k_BT}. \tag{72}$$

D. Markovian limit

The Markovian approximation to the non-Markovian Langevin coefficients is obtained by taking the limit as ω goes to zero. For the stochastic force correlation function, we take $\omega = 0$ directly in Eq.(68), from which we define the Markovian stochastic force correlation function as

$$\tilde{D}(x,0) = \sum_{\alpha\in\{L,R\}} D^{\text{eq.}}_{\alpha}(x) + D^{\text{neq.}}(x). \tag{73}$$

$D^{\text{eq.}}_{\alpha}(x)$ is the correlation function for the stochastic force processes due to the system interacting only with the α lead, and it is given by

$$D^{\text{eq.}}_{\alpha}(x) = -\frac{1}{2\pi}\int_{-\infty}^{\infty} d\epsilon k_B T_\alpha \frac{\partial f_\alpha}{\partial \epsilon} \sum_{\beta\in\{L,R\}} \text{Tr}\Big\{\frac{dh_{\text{ad.}}(x)}{dx} A_\alpha(x,\epsilon)\frac{dh_{\text{ad.}}(x)}{dx} A_\beta(x,\epsilon)\Big\} .$$

$D^{\text{neq.}}(x)$ is the correlation function for the stochastic force processes arising due to nonequilibrium electron transport from one lead to the other,

$$D^{\text{neq.}}(x) = \frac{1}{2\pi}\int_{-\infty}^{\infty} d\epsilon \, \Big(f_L(\epsilon) - f_R(\epsilon)\Big)^2 \text{Tr}\Big\{\frac{dh_{\text{ad.}}(x)}{dx} A_L(x,\epsilon)\frac{dh_{\text{ad.}}(x)}{dx} A_R(x,\epsilon)\Big\} .$$

To derive the Markovian friction, we take Eq.(67) and take an expansion in small ω,

$$A(x,\epsilon \pm \frac{\omega}{2}) \approx A(x,\epsilon) \pm \frac{\omega}{2}\frac{\partial A(x)}{\partial \epsilon}, \qquad f(\epsilon \pm \frac{\omega}{2}) \approx f(\epsilon) \pm \frac{\omega}{2}\frac{\partial f}{\partial \epsilon}. \tag{74}$$

The terms proportional to $1/\omega$ cancel out exactly, and the Markovian friction corresponds to the ω independent term, given as

$$\tilde{\gamma}(x,0) = \sum_{\alpha\in\{L,R\}} \gamma^{\text{eq.}}_{\alpha}(x) + \gamma^{\text{neq.}}(x). \tag{75}$$

The component of the friction due to interaction with lead α is

$$\gamma^{\text{eq.}}_{\alpha}(x) = -\frac{1}{4\pi}\int_{-\infty}^{\infty} d\epsilon \frac{\partial f_\alpha}{\partial \epsilon} \sum_{\beta\in\{L,R\}} \text{Tr}\Big\{\frac{dh_{\text{ad.}}(x)}{dx} A_\alpha(x,\epsilon)\frac{dh_{\text{ad.}}(x)}{dx} A_\beta(x,\epsilon)\Big\}, \tag{76}$$

while the nonequilibrium component due to electron transport across the junction is

$$\gamma^{\text{neq.}}(x) = \frac{1}{4\pi}\int_{-\infty}^{\infty} d\epsilon \, \Big(f_L(\epsilon) - f_R(\epsilon)\Big) \text{Tr}\Big\{\frac{dh_{\text{ad.}}(x)}{dx}\frac{\partial A_R(x)}{\partial \epsilon}\frac{dh_{\text{ad.}}(x)}{dx} A_L(x,\epsilon) \; - \frac{dh_{\text{ad.}}(x)}{dx} A_R(x,\epsilon)\frac{dh_{\text{ad.}}(x)}{dx}\frac{\partial A_L(x)}{\partial \epsilon}\Big\}. \tag{77}$$

It is immediately clear that $\gamma^{\text{eq.}}_{\alpha}(x)$ and $D^{\text{eq.}}_{\alpha}(x)$ satisfy the fluctuation-dissipation relation according to

$$D^{\text{eq.}}_{\alpha}(x) = 2k_B T_\alpha \gamma^{\text{eq.}}_{\alpha}(x). \tag{78}$$

III. QUANTUM TRANSPORT METHODOLOGY

In order to benchmark the various mixed quantum-classical methods used in this work, we employed the hierarchical equations of motion (HEOM) approach. The HEOM approach is a quantum master equation derived from the Feynman-Vernon influence functional that treats all degrees of freedom, both electronic and vibrational, fully quantum mechanically and numerically exactly. Given that the underlying theory of the HEOM approach is quite extensive and it is well documented in Refs. [12–22], we will only provide a brief outline here.

A. Further Details about the Molecule-Lead Coupling

The explicit form of the leads' Hamiltonian as well as the molecule-leads coupling is given in the main text. In this subsection, supplement the model with extra details required for numerical simulation. First, in all calculations, it is assumed that the molecule and leads are initially uncoupled: $\rho(0) = \rho_{\rm mol}(0) \otimes \rho_{\rm leads}(0)$. Given the local equilibrium states of each lead, this implies that

$$\rho_{\rm leads}(0) = \prod_{\alpha} \frac{e^{-(H_{{\rm leads},\alpha}-\mu_\alpha)/k_{\rm B}T}}{{\rm Tr}_{{\rm leads},\alpha}\left[e^{-(H_{{\rm leads},\alpha}-\mu_\alpha)/k_{\rm B}T}\right]}. \tag{79}$$

For times $t > 0$, the molecule and leads interact via $H_{\text{mol-leads}}$, with the interaction further characterized by the spectral density of lead α,

$$\Gamma_{\alpha,mm'}(E) = 2\pi \sum_k V_{k\alpha,m} V^*_{k\alpha,m'} \delta(E - \varepsilon_k). \tag{80}$$

To mimic the wide-band limit used for the electronic forces in the mixed quantum-classical simulations, the quantum simulations used a Lorentzian spectral density

$$\Gamma_{\alpha,mm'}(E) = V_{\alpha,m} V^*_{\alpha,m} \frac{W_\alpha}{(E-\mu_\alpha)^2 + W_\alpha^2}, \tag{81}$$

which has a single peak centered around the chemical potential, μ_α, and bandwidth W_α, and set a large bandwidth of $W_\alpha = 25$ eV. Here, the quantities $V_{\alpha,m}$ represent a constant coupling strength between lead α and state m. These quantities form the molecule-lead coupling strength, $\Gamma_{\alpha,mm'} = 2\pi V_{\alpha,m} V^*_{\alpha,m'}$, which is diagonal in the electronic states given the chain nature of the model:

$$\Gamma_{\alpha,12} = \Gamma_{\alpha,21} = \Gamma_{R,11} = \Gamma_{L,22} = 0. \tag{82}$$

The remaining molecule-lead couplings are set to the same value, $\Gamma_{R,22} = \Gamma_{L,11} = \Gamma_L = \Gamma_R = \Gamma$.

B. Hierarchical Equations of Motion

Similar to the procedure to obtain classical equations of motion presented in Sec. I, the HEOM approach also relies on the Feynman-Vernon influence functional. However, unlike in Sec. I, the object of interest is the reduced density operator of the molecule, $\rho_{\rm mol}(t) = {\rm Tr}_{\rm leads}\{\rho_{\rm tot.}(t)\}$, such that the influence functional now contains the effective action of the leads on the molecule. By exploiting the fact that the molecule-lead coupling Hamiltonian is linear in lead operators and that $H_{\rm leads}$ is noninteracting and quadratic, the influence functional can be written exactly in terms of its second-order cumulant and is completely characterized by the two-time correlation functions

$$C^\sigma_{\alpha,mm'}(t-\tau) = \sum_k V^\sigma_{k\alpha,m} V^{\bar\sigma}_{k\alpha,m'} {\rm Tr}_{\rm leads}\left[c^\sigma_{k\alpha}(t) c^{\bar\sigma}_{k\alpha}(\tau) \rho_{\rm leads}(0)\right]. \tag{83}$$

Here, new notation has been introduced to condense the lead operators: $\sigma = \pm$ and $\bar\sigma = \mp$, with $c^-_{k\alpha} = c_{k\alpha}$, $c^+_{k\alpha} = c^\dagger_{k\alpha}$, $V^+_{k\alpha,m} = V_{k\alpha,m}$, and $V^-_{k\alpha,m} = V^*_{k\alpha,m}$. These have additionally been written in the interaction picture with respect to the leads' degrees of freedom has been introduced: $c^\sigma_{k\alpha}(t) = e^{iH_{\rm leads}t} c^\sigma_{k\alpha} e^{-iH_{\rm leads}t}$.

Furthermore, using the spectral density of the leads introduced above, the two-time lead correlation functions can be rewritten as

$$C^\sigma_{\alpha,mm'}(t-\tau) = \frac{1}{2\pi}\int_{-\infty}^{\infty} dE\, e^{i\sigma E(t-\tau)} \Gamma_{\alpha,mm'}(E) f^\sigma_\alpha(E), \tag{84}$$

which describes the occupation of electrons for $\sigma = +$ and holes for $\sigma = -$. Via a direct fitting of $C^\sigma_{\alpha,mm'}(t-\tau)$ in the time-domain [23], the two-time lead correlation functions can then be expanded as a sum of exponential functions,

$$C^\sigma_{\alpha,mm'}(t-\tau) = V_{\alpha,m} V^*_{\alpha,m'} \sum_{\ell=0}^{\ell_{\rm max}} \eta_{\alpha,\sigma,\ell,m} e^{-\kappa_{\alpha,\sigma,\ell,m} t}. \tag{85}$$

At room temperature and for the Lorentzian spectral density given above, six terms were sufficient to converge the accuracy of the decomposition.

With this exponential decomposition, the influence functional can then be rewritten as a set of coupled linear equations of motion between the molecular density matrix, $\rho_{\text{mol}}(t)$, and a series of auxiliary density operators (ADOs) of various tiers, $\rho^{(n)}$. For time-independent Hamiltonians, this can then be concisely expressed as a linear, time-local quantum master equation for the density matrix-like operator $\boldsymbol{\rho}$:

$$\frac{\partial}{\partial t}\boldsymbol{\rho}(t) = \mathcal{L}_{\text{HEOM}}\boldsymbol{\rho}(t). \tag{86}$$

where $\boldsymbol{\rho}(t)$ contains the molecular density matrix and all ADOs,

$$\boldsymbol{\rho}(t) = \left[\rho_{\text{mol}}, \rho^{(1)}_{j_1}, \rho^{(1)}_{j_2}, \ldots, \rho^{(2)}_{\mathbf{j}^{(2)}_k}, \ldots, \rho^{(N_{\max})}_{\mathbf{j}^{(N_{\max})}_k}\right]^T. \tag{87}$$

The ADOs are operators of the same dimension as ρ_{mol}, except that instead of describing the state of the molecule, they contain all information about the influence of the leads on the molecular dynamics, including all higher-order transport and nonmarkovian effects. Although the number of ADOs and their indexing initially appears complicated, they arise naturally from HEOM theory. Essentially, interactions between the molecule and the leads are represented by a series of virtual fermionic levels, where the kth virtual level is described by the super-index $j_k = (\alpha_k, \sigma_k, \ell_k, m_k)$. This refers to an effective interaction between level m_k in the molecule and lead α_k, in which an electron is created, $\sigma_k = +$, or destroyed, $\sigma_k = -$, with energy related to $\gamma_{\alpha_k,\sigma_k,\ell_k}$. Such first-order interactions are described by the 1st-tier ADOs, $\rho^{(1)}_{j_k}$, where the tier number $(n) = (1)$ refers to the number of virtual fermionic levels contained in the interaction described by this ADO. The number of 1st-tier ADOs is $2N_{\text{leads}}N_m N_\ell$.

Following this description, higher-order interaction effects are naturally described by higher-tier ADOs, $\rho^{(n)}_{\mathbf{j}^{(n)}_k}$ with $(n) > (1)$. The vector $\mathbf{j}^{(n)}_k = (j_{n_k}, \ldots, j_{2_k}, j_{1_k})$ now contains n virtual fermionic levels, each of which can be any of the $2N_{\text{leads}}N_m N_\ell$ levels outlined above. The time evolution of ρ_{mol} and the ADOs is contained in the time-local superoperator $\mathcal{L}_{\text{HEOM}}$, which is constructed from the equation of motion for each ADO:

$$\frac{\partial}{\partial t}\rho^{(n)}_{\mathbf{j}^{(n)}_k} = -i\left[H_{\text{mol}}, \rho^{(n)}_{\mathbf{j}^{(n)}_k}\right] - \sum_{r=1}^{n}\kappa_{j_{r_k}}\rho^{(n)}_{\mathbf{j}^{(n)}_k} - i\sum_{r=1}^{n}(-1)^{n-r}\mathcal{C}_{j_{r_k}}\rho^{(n-1)}_{\mathbf{j}^{(n),-}_{k,r}} - i\sum_{j_r}\mathcal{A}_{\bar{j}_r}\rho^{(n+1)}_{\mathbf{j}^{(n),+}_{k,r}}. \tag{88}$$

One obtains the equation of motion for ρ_{mol} by setting $n = 0$ in Eq.(88). In Eq.(88), we have introduced the superoperators coupling different tiers of the hierarchy,

$$\mathcal{C}_{j_r}\rho^{(n)}_{\mathbf{j}^{(n)}_k}(t) = V_{\alpha_r,m_r}\left(\eta_{j_r} d^{\sigma_r}_{m_r}\rho^{(n)}_{\mathbf{j}^{(n)}_k}(t) - (-1)^n \eta^*_{\bar{j}_r}\rho^{(n)}_{\mathbf{j}^{(n)}_k}(t) d^{\sigma_r}_{m_r}\right), \tag{89}$$

$$\mathcal{A}_{\bar{j}_r}\rho^{(n)}_{\mathbf{j}^{(n)}_k}(t) = V_{\alpha,m}\left(d^{\bar{\sigma}}_m\rho^{(n)}_{\mathbf{j}^{(n)}_k}(t) + (-1)^n\rho^{(n)}_{\mathbf{j}^{(n)}_k}(t) d^{\bar{\sigma}}_m\right), \tag{90}$$

as well as three further super-indices: $\mathbf{j}^{(n),-}_{k,r} = (j_{n_k}, \ldots, j_{r_k-1}, j_{r_k+1}, \ldots, , j_{r_1})$, $\mathbf{j}^{(n),+}_{k,r} = (j_r, j_{n_k}, \ldots, j_{1_k})$, and $\bar{j}_r = (\alpha_r, \bar{\sigma}_r, \ell_r, m_r)$.

Eq.(86) can be solved by direct time propagation, where the initial condition for a separated molecule and leads is $\boldsymbol{\rho}(0) = [\rho_{\text{mol}}(0), 0, \ldots, 0]$ [24]. In this work, we are interested in observables solely in the nonequilibrium steady state. To this end, instead of propagating Eq.(86), we calculate $\boldsymbol{\rho}^{\text{ss}}$ directly via an iterative Krylov space method [25].

Beyond the method used to calculate the steady state, there are other numerical details required for HEOM. First, although the hierarchy naturally terminates at $n = 2N_{\text{leads}}N_m N_\ell$, the expectation values of lower-order observables such as vibrational excitation and electric current often converge when the equations of motion are truncated at a much lower tier, $N_{\max}$. Second, the molecular dynamics also crucially depends on the number of vibrational basis states used to represent the harmonic vibrational mode, $N_{\text{bas.}}$. In practice, n and $N_{\text{bas.}}$ are chosen by starting with some initial guess and increasing the values until the observables converge within a tolerance. In this work, we found that expectation values of all the observables of interest converged at $n = 2$.

A further convergence parameter is the number of vibrational basis states used to represent the vibrational degrees of freedom, $N_{\text{bas.}}$. In this work, we exclusively worked in the occupation number representation,

$$b = \frac{1}{\sqrt{2}}\left(\hat{x} + i\hat{p}\right) \qquad ; \qquad b^\dagger = \frac{1}{\sqrt{2}}\left(\hat{x} - i\hat{p}\right), \tag{91}$$

where $b^\dagger$ and b create and annihilate a vibrational phonon with energy Ω, respectively. In this basis, the molecular Hamiltonian can be written as

$$H_{\mathrm{mol}} = \Delta\left(d_1^\dagger d_1 - d_2^\dagger d_2\right) + \lambda\left(d_1^\dagger d_2 + d_2^\dagger d_1\right)\left(b + b^\dagger\right) + \frac{\Omega}{2} b^\dagger b, \tag{92}$$

when neglecting the zero-point energy, as it does not affect the dynamics. In this basis, all observables of interest converged at $N_{\mathrm{bas.}} = 100$.

C. Observables in HEOM

Via the HEOM approach, one can calculate the expectation values of all observables in the molecule and lead. In this work, we are mainly interested in the average excitation of the vibrational mode, $\langle N_{\mathrm{vib}}\rangle$. We examine these observables exclusively in the nonequilibrium steady state, $\boldsymbol{\rho}^{\mathrm{ss}}$, where $\mathcal{L}_{\mathrm{HEOM}}\boldsymbol{\rho}^{\mathrm{ss}} = \mathbf{0}$.

Given that the vibrational number operator $N_{\mathrm{vib}} = b^\dagger b = \frac{1}{2}\left(\hat{x}^2 + \hat{p}^2\right)$ exists only in the molecular Hilbert space, it can be calculated from the molecular density matrix alone:

$$\langle N_{\mathrm{vib}}\rangle = \mathrm{Tr}_{\mathrm{mol}}\left[b^\dagger b \rho_{\mathrm{mol}}^{\mathrm{ss}}\right]. \tag{93}$$

[1] F. Chen, K. Miwa, and M. Galperin, Current-induced forces for nonadiabatic molecular dynamics, J. Phys. Chem. A **123**, 693 (2019).
[2] J.-T. Lü, M. Brandbyge, P. Hedegård, T. N. Todorov, and D. Dundas, Current-induced atomic dynamics, instabilities, and raman signals: Quasiclassical langevin equation approach, Phys. Rev. B **85**, 245444 (2012).
[3] T. N. Todorov, D. Dundas, A. T. Paxton, and A. P. Horsfield, Nonconservative current-induced forces: A physical interpretation, Beilstein J. Nanotechnol. **2**, 727 (2011).
[4] T. N. Todorov, D. Dundas, J.-T. Lü, M. Brandbyge, and P. Hedegård, Current-induced forces: a simple derivation, Eur. J. Phys. **35**, 065004 (2014).
[5] R. J. Preston, M. F. Gelin, and D. S. Kosov, First-passage time theory of activated rate chemical processes in electronic molecular junctions, J. Chem. Phys. **154**, 114108 (2021).
[6] F. Chen, K. Miwa, and M. Galperin, Electronic friction in interacting systems, J. Chem. Phys. **150**, 174101 (2019).
[7] W. Dou and J. E. Subotnik, Universality of electronic friction: Equivalence of von oppen's nonequilibrium green's function approach and the head-gordon–tully model at equilibrium, Phys. Rev. B **96**, 104305 (2017).
[8] S. L. Rudge, Y. Ke, and M. Thoss, Current-induced forces in nanosystems: A hierarchical equations of motion approach, Phys. Rev. B **107**, 115416 (2023).
[9] W. Dou, G. Miao, and J. E. Subotnik, Born-oppenheimer dynamics, electronic friction, and the inclusion of electron-electron interactions, Phys. Rev. Lett. **119**, 046001 (2017).
[10] S. L. Rudge, C. Kaspar, R. L. Grether, S. Wolf, G. Stock, and M. Thoss, Nonadiabatic dynamics of molecules interacting with metal surfaces: A quantum–classical approach based on langevin dynamics and the hierarchical equations of motion, J. Chem. Phys. **160**, 184106 (2024).
[11] M. Sachs, B. Leimkuhler, and V. Danos, Langevin dynamics with variable coefficients and nonconservative forces: From stationary states to numerical methods, Entropy **19**, 647 (2017).
[12] Y. Tanimura and R. Kubo, Time evolution of a quantum system in contact with a nearly gaussian-markoffian noise bath, J. Phys. Soc. Jpn. **58**, 101 (1989).
[13] Y. Tanimura, Stochastic liouville, langevin, fokker–planck, and master equation approaches to quantum dissipative systems, J. Phys. Soc. Jpn **75**, 082001 (2006).
[14] Y. Tanimura, Numerically "exact" approach to open quantum dynamics: The hierarchical equations of motion (heom), J. Chem. Phys. **153**, 020901 (2020).
[15] R. Härtle, G. Cohen, D. R. Reichman, and A. J. Millis, Transport through an anderson impurity: Current ringing, nonlinear magnetization, and a direct comparison of continuous-time quantum monte carlo and hierarchical quantum master equations, Phys. Rev. B **92**, 085430 (2015).
[16] J. Jin, S. Welack, J. Luo, X.-Q. Li, P. Cui, R.-X. Xu, and Y. Yan, Dynamics of quantum dissipation systems interacting with fermion and boson grand canonical bath ensembles: Hierarchical equations of motion approach, J. Chem. Phys. **126**, 134113 (2007).
[17] J. Jin, X. Zheng, and Y. Yan, Exact dynamics of dissipative electronic systems and quantum transport: Hierarchical equations of motion approach, J. Chem. Phys. **128**, 234703 (2008).
[18] C. Schinabeck, R. Härtle, and M. Thoss, Hierarchical quantum master equation approach to electronic-vibrational coupling in nonequilibrium transport through nanosystems: Reservoir formulation and application to vibrational instabilities, Phys. Rev. B **97**, 235429 (2018).
[19] X. Zheng, J. Jin, S. Welack, M. Luo, and Y. Yan, Numerical approach to time-dependent quantum transport and dynamical kondo transition, J. Chem. Phys. **130**, 164708 (2009).
[20] Y. Yan, Theory of open quantum systems with bath of electrons and phonons and spins: Many-dissipaton density matrixes approach, J. Chem. Phys. **140**, 054105 (2014).
[21] S. Wenderoth, J. Bätge, and R. Härtle, Sharp peaks in the conductance of a double quantum dot and a quantum-dot spin valve at high temperatures: A hierarchical quantum master equation approach, Phys. Rev. B **94**, 121303(R) (2016).
[22] L. Ye, X. Wang, D. Hou, R.-X. Xu, X. Zheng, and Y. Yan, Heom-quick: a program for accurate, efficient, and universal characterization of strongly correlated quantum impurity systems, WIREs Comput. Mol. Sci. **6**, 608 (2016).
[23] H. Takahashi, S. Rudge, C. Kaspar, M. Thoss, and R. Borrelli, High accuracy exponential decomposition of bath correlation functions for arbitrary and structured spectral densities: Emerging methodologies and new approaches, J. Chem. Phys. **160**, 204105 (2024).
[24] C. Schinabeck, A. Erpenbeck, R. Härtle, and M. Thoss, Hierarchical quantum master equation approach to electronic-vibrational coupling in nonequilibrium transport through nanosystems, Phys. Rev. B **94**, 201407(R) (2016).
[25] C. Kaspar and M. Thoss, Efficient steady-state solver for the hierarchical equations of motion approach: Formulation and application to charge transport through nanosystems, J. Phys. Chem. A **125**, 5190 (2021).
